# Bacterial Viability and Physical Properties of Antibacterially Modified Experimental Dental Resin Composites

**DOI:** 10.1371/journal.pone.0079119

**Published:** 2013-11-01

**Authors:** Stefan Rüttermann, Taina Trellenkamp, Nora Bergmann, Thomas Beikler, Helmut Ritter, Ralf Janda

**Affiliations:** 1 Heinrich-Heine-University, Medical Faculty, Centre of Dentistry, Department of Operative Dentistry, Periodontology and Endodontics, Düsseldorf, Germany; 2 Heinrich-Heine-University, Institute of Organic Chemistry and Macromolecular Chemistry, Düsseldorf, Germany; University Hospital of the Albert-Ludwigs-University Freiburg, Germany

## Abstract

**Purpose:**

To investigate the antibacterial effect and the effect on the material properties of a novel delivery system with Irgasan as active agent and methacrylated polymerizable Irgasan when added to experimental dental resin composites.

**Materials and Methods:**

A delivery system based on novel polymeric hollow beads, loaded with Irgasan and methacrylated polymerizable Irgasan as active agents were used to manufacture three commonly formulated experimental resin composites. The non-modified resin was used as standard (ST). Material A contained the delivery system providing 4 % (m/m) Irgasan, material B contained 4 % (m/m) methacrylated Irgasan and material C 8 % (m/m) methacrylated Irgasan. Flexural strength (FS), flexural modulus (FM), water sorption (WS), solubility (SL), surface roughness R_a_, polymerization shrinkage, contact angle Θ, total surface free energy γ_S_ and its apolar γ_S_^LW^, polar γ_S_^AB^, Lewis acid γ_S_^+^and base γ_S_^-^ term as well as bacterial viability were determined. Significance was p < 0.05.

**Results:**

The materials A to C were not unacceptably influenced by the modifications and achieved the minimum values for FS, WS and SL as requested by EN ISO 4049 and did not differ from ST what was also found for R_a_. Only A had lower FM than ST. Θ of A and C was higher and γ_S_^AB^ of A and B was lower than of ST. Materials A to C had higher γ_S_^+^ than ST. The antibacterial effect of materials A to C was significantly increased when compared with ST meaning that significantly less vital cells were found.

**Conclusion:**

Dental resin composites with small quantities of a novel antibacterially doped delivery system or with an antibacterial monomer provided acceptable physical properties and good antibacterial effectiveness. The sorption material being part of the delivery system can be used as a vehicle for any other active agent.

## Introduction

Several attempts have been made to modify dental resin composites to avoid or at least to diminish pellicle and bacterial adherence which is considered to be important in early plaque formation [1-7]. Mainly two concepts were investigated to reduce bacterial adherence: (a) alteration of the resin matrix by adding fluoride-releasing materials [8], silver nanoparticles [9], fluorine polymers [10], antimicrobial monomers, polymers or additives [11-14] or quaternary ammonium polyethylenimine nanoparticles [15,16], (b) reduction of the materials’ wettabilities since some evidence was found that materials with low wettability, meaning low surface free energy (SFE), resulted in significantly less bacterial adherence and thus less plaque [10,17-23]. But solely quaternary ammonium polyethylenimine nanoparticles of concept (a) were reported to have strong antibacterial activity without affecting flexural strength and modulus of dental resin composites [15,16]. Also concept (b) was challenged by other studies reporting no strong correlation of streptococcal adhesion and substratum surface roughness or SFE, respectively [23-25], and found that SFE’s influence on bacterial adhesion significantly decreased after saliva coating [23,26].

More recent literature presented an entirely new approach to obtain dental resin composites with low SFE, antimicrobial effect and acceptable physical properties [4,5]. Novel polymer hollow beads (Poly-Pore, Table 1) used as a carrier material, were highly loaded with different types of low surface tension agents resulting in a delivery system which was added in small quantities to experimental resin-based restorative materials. It was hypothesized and found that (a) single delivery particles were always present in the outer material’s surface, (b) occurring abrasion processes, simulated by polishing procedures, destroyed the delivery particles, and (c) the low surface tension agents flushed the surface and thus reduced the material’s SFE.

**Table 1:** Raw material.

Code	Product / properties	Batch	Company
Photoini	α.α-dimethoxy-α-phenylacetophenone	0066162S	Ciba Specialities Chemical Inc.. Basel. Switzerland
Stab	pentaerythrityl-tetrakis[3-(3,5-di-tert.-butyl-4-hydroxyphenyl)-propionate	26099IC3	Ciba Specialities Chemical Inc.. Basel. Switzerland
TTEGDMA	tetraethyleneglycole dimethacrylate, standard monomer, functionality=2, MW=330 [g mol^-1^], good chemical and physical properties, very low viscosity (14 Pa s, 25 °C), diluting	J1620	Cray Valley. Paris. France
UV-Stab	2-hydroxy-4-methoxy-bezophenone	411351/143302	Fluka. Buchs. Switzerland
UDMA	7.7.9-trimethyl-4,13-dioxo-3,14-dioxa-5,12-diaza-hexadecan-1,16-diol-dimethacrylate, standard monomer, functionality=2, MW=471 [g mol^-1^], flexible, tough, very good chemical resistance, medium viscosity (10000 mPa s, 25 °C)	330503057	Rahn AG. Zürich. Switzerland
Bis-GMA	Bis-GMA, standard monomer, functionality=2, MW=513 [g mol^-1^], rigid, very good chemical resistance, very high viscosity (4500 mPa s, 60 °C)	2008218303	Rahn AG. Zürich. Switzerland
CQ	D,L-camphorquinone	0148990002	Rahn AG. Zürich. Switzerland
Amine	ethyl-4-(dimethylamino)-benzoate	310170	Rahn AG. Zürich. Switzerland
Glass	strontiumborosilicate glass (Glass G0 18-093, 0.7 µm). silaned (3-methacryloyloxypropyltrimethoxy silane), D=2.6 [g cm^-3^]	Lab14701	Schott Electronic Packaging GmbH. Landshut. Germany
Poly-	Poly-Pore, cross-linked polyallyl methacrylate, adsorber, hollow beads, diameter 20 - 40 µm	L07070303AB	AMCOL Health & beauty Solutions, Arlington Heights, IL, USA
Irga	Irgasan, 5-chloro-2-(2,4-dichlorophenoxy)phenole	1124816	Sigma Aldrich GmbH, Steinheim, Germany
Methacryl-Irga	5-chloro-2-(2,4-dichlorophenoxy)phenyl methacrylate		university laboratory
Poly-Irga	loaded with 80% Irgasan, D=1.0 [g cm^-3^]	experimental products	university laboratory

Information is based on the manufacturers’ technical data sheets

The goal of the present investigation was, based on the new concepts of the aforesaid literature [4,5], to examine the material properties (flexural strength, modulus, water sorption, solubility, surface roughness, polymerization shrinkage, contact angle, surface free energy) of four antibacterially modified experimental dental resin composites and the bacterial viability (*A. naeslundii, A. viscosus*, *S.* oralis, *S.* mitis, *S.* sanguinis) after 8 or 24 hours, respectively, on these materials. One material contained the polymer hollow beads loaded with the antibacterially effective Irgasan (5-chloro-2-(2,4-dichlorophenoxy)phenol, Table 1), two materials were modified with different portions of methacrylated Irgasan (5-chloro-2-(2,4-dichlorophenoxy)phenyl methacrylate, Table 1) and the unmodified material was used as the standard ST. 5-chloro-2-(2,4-dichlorophenoxy)phenol (tradenames: Irgasan, Triclosan) is a well-known and well-proven broad spectrum antimicrobial agent. It inhibits the enoyl-acyl-carrier protein reductase component of type II fatty acid synthase in bacteria, the mammalian fatty acid synthase and provides anticariogenic activity [27-29]. The null hypothesis was that the materials did not differ from ST or among each other (a) in the materials properties and (b) in the total bacterial counts or in the respective bacterium’s viability after 8 or 24 hours observation time.

## Materials and Methods

**Figure 1:**
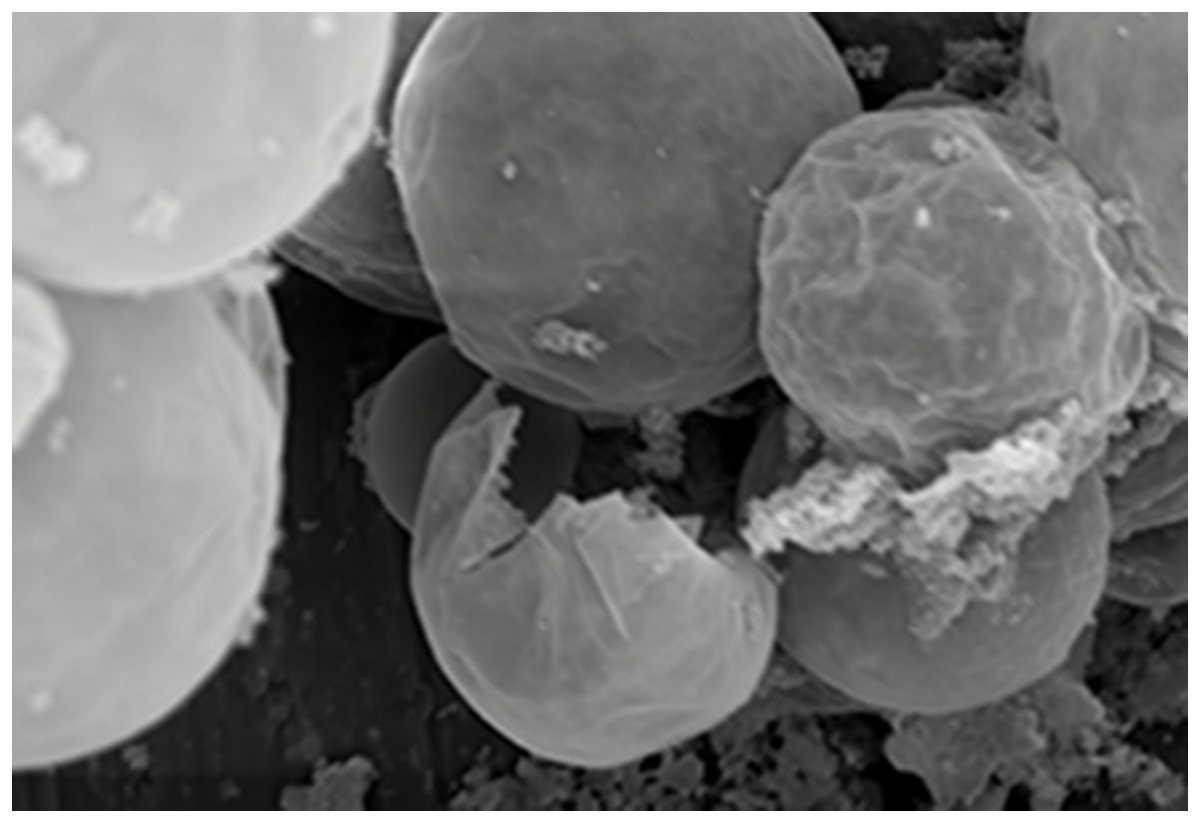
Poly-Pore hollow bead sorption material, unloaded (magnification 500 x).

**Figure 2:**
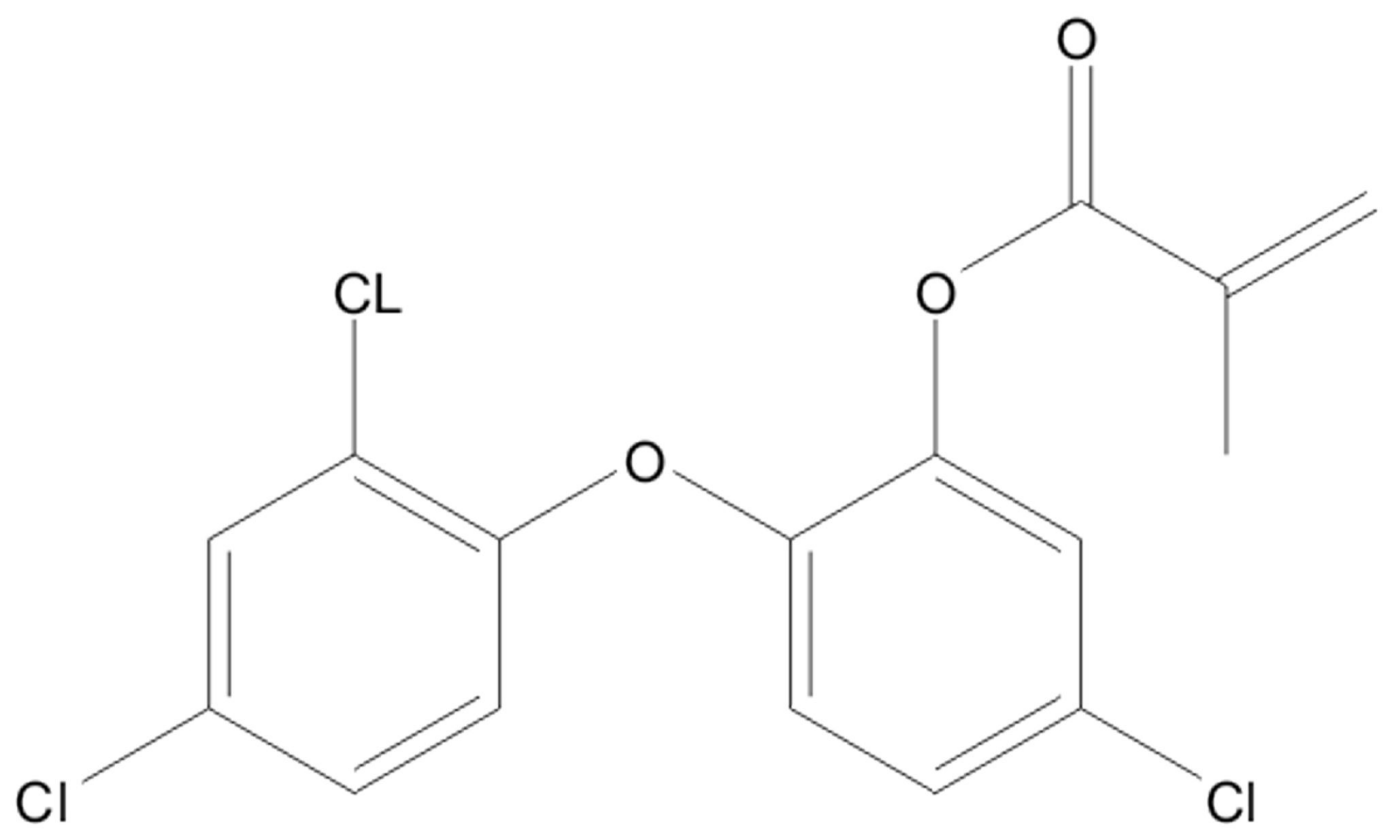
Chemical formula of Methacryl-Irga.

Four experimental resin-based restorative materials were prepared (Tables 1 and 2) using a laboratory vacuum planet kneader (Herbst Maschinenfabrik GmbH, Buxtehude, Germany). The standard ST represented a common formulation for resin-based restorative materials. Poly-Pore sorption material loaded with Irgasan (5-chloro-2-(2,4-dichlorophenoxy)phenole) as active agent (Tables 1 and 2, Figure 1) was the delivery system. ST was modified by replacing parts of the glass filler with the delivery system resulting in material A. The matrix of ST was partly replaced by polymerizable Methacryl-Irga (Table 1 and 2, Figure 2) to obtain materials B and C. Flexural strength, flexural modulus, water sorption, solubility and surface roughness R_a_ were determined. Curing was done with a quartz-tungsten halogen device (Spectrum 800, Dentsply De Trey GmbH, Constance, Germany) performing an irradiance of 931 ± 90 mW cm-2 which was checked periodically with the bluephase meter (Ivoclar Vivadent AG, Schaan, Liechtenstein). 

**Table 2:** Formulations of experimental resin-based restorative materials (ST = standard).

Experimental resin-based filling materials. formulations [weight-%]				
ST	A	B	C	Raw material
73.00	68.00	73.00	73.00	Glass
---	5.00	---	---	Poly-Irga
---	---	4.00	---	Methacryl-Irga
---	---	---	8.00	Methacryl-Irga
27.00	27.00	23.00	19.00	Matrix
0	4	4	8	active agent

Matrix: UDMA=44.10, Bis-GMA=30.00, TTEGDMA=25.00, photonitiator=0.30, CQ=0.20, amine=0.10, stabilizer=0.10

### Manufacturing of the delivery system

Irgasan (Table 1) was dissolved in great excess of butanone (Lot 244238, Brenntag GmbH, Mülheim, Germany) and the Poly-Pore sorption material (Table 1) was added while stirring. The obtained thin slurry was stirred for approximately 10 minutes to optimally wet the sorption particles. Next, while stirring, the mixture was slightly warmed to evaporate the solvent. Stirring was stopped when the mixture became too stiff and it was put in a drying closet at 50 °C until constant weight was obtained (generally 24 hours). After this treatment the loaded sorption material, representing the delivery system, appeared totally dry and powdery.

### Analytical data

^1^H-NMR (CDCl_3_): δ = 1.94 (t, ^3^J(H,H) = 1.28 Hz, 3H, methyl -CH_3_); 5.69 (q, ^3^J(H,H) = 1.47 Hz, 1H, acryl =CH_2_); 6.18 (t, ^3^J(H,H) = 1.05 Hz, 1H, acryl =CH_2_); 6.86 (dd, ^3^J(H,H) = 8,73 Hz, 1H, aryl CH); 6.89 (d, ^3^J(H,H) = 8,73 Hz, 1H, aryl); 7.14 (dd, ^3^J(H,H) = 8.73 Hz, ^4^J(H,H) = 2.47 Hz, 1H, aryl); 7.18 (dd, ^3^J(H,H) = 8.73 Hz, ^4^J(H,H) = 2.47 Hz, 1H, aryl); 7.25 (d, ^3^J(H,H) = 2.33 Hz, 1H, aryl); 7.41 (d, ^3^J(H,H) = 2.46 Hz, 1H, aryl) ppm.

^13^C-NMR (CDCl_3_): δ = 18.21 (methyl -CH_3_); 119.91 (aryl); 120.66 (aryl); 124.59 (acryl =CH_2_); 125.69 (aryl C-Cl); 126.93 (aryl); 128.03 (aryl); 128.1 (aryl); 129.23 (aryl C-Cl); 129.46 (aryl C-Cl); 130.33 (aryl); 134.81 (acryl C=C); 142.06 (aryl -ether C-O); 146.5 (aryl -ester C-O); 151.05 (aryl -ether C-O); 164.61 (ester C=O) ppm.

FT-IR (diamond): ν = 3092 (w, =CH_2_), 2983 (w, CH), 2875 (w, CH), 1741 (ss, C=O), 1636 (ss, C=C), 1098 (s, C-O-C) cm^-1^.

GC/MS m/z (%): 361 (3), 360 (20), 358 (56), 357 (8), 356 (60) [M^+^], 252 (20), 189 (10), 69 (100), 41 (14).

### Flexural strength and flexural modulus

Ten specimens (25 ± 2 x 2 ± 0.1 x 2 ± 0.1 mm) were made from each material according to ISO 4049 [33] and cured in five 40 s increments from each side (400 s in total). Testing was done after 24 hours of water storage at 37 ° with the three-point-bending test (universal testing machine, crosshead speed of 0.75 mm min^-1^, Model 106.L, Test GmbH, Erkrath, Germany).

Flexural strength was calculated by σ = (3FL) / (2bh^2^) and flexural modulus by E = (L^3^/ 4bh^3^) x (F/Y) both expressed in MPa with F = maximum strength, L = distance between the rests, b = width of the specimen, h = height of the specimen, and F/Y = slope of the linear part of the stress-strain curve.

### Water sorption and solubility

Ten disks (thickness: 1 ± 0.1 mm, diameter: 15 ± 0.1 mm) were made from each material according to ISO 4049 [33] and cured in eight overlapping steps of 40 s each side (320 s in total). The specimens were dried and weighed (analytical balance, Toledo XS, Mettler Toledo GmbH, Greifensee, Switzerland) until mass m_1_ was constant. Their volumes V were determined by measuring their diameters and thicknesses. After 7 days water storage at 37 ± 1 °C mass m_2_ was weighed. Then the specimens were totally re-dried to obtain mass m_3_. Water sorption was calculated by W_sp_ = (m_2_ - m_3_) / V and solubility by W_sl_ = (m_1_ - m_3_) / V both expressed in µg mm^-3^.

### Surface roughness R_a_

After contact angle measurement the specimens were polished again and their surface roughness R_a_ was determined (Surftest SJ-210 profilometer, diamond pick-up, tip radius: 5 µm, load: 4 mN, Mitutoyo Corporation, Kawasaki, Japan). Each specimen was measured five times at different distances and in different directions (evaluation length of 0.8 mm, stylus speed 0.5 mm s^-1^) from its center. For each measurement, the stylus was automatically moved five times forward and backward along the same path. The data were filtered with a cut-off (Λc) of 0.8 mm (Gauss profile-filter) following DIN EN ISO 4288 [34]. 

### Polymerization shrinkage

Polymerization shrinkage was determined according to the Archimedes’ principle (Density Determination Kit, Mettler Toledo GmbH, Gießen, Germany). After weighing the specimens in air and in water, their densities were calculated in g cm-3 by D = (A/(A-B)) x (D0-DL) + DL with D = density of sample, A = weight of sample in air, B = weight of sample in water, D0 = density of water, and DL = air density (0.0012 g cm-3). Firstly the density D1 of the uncured material (sphere-shaped, approximately 0.1 g) was determined. Next, disks (diameter 10 ± 0.1 mm, thickness 1 ± 0.1 mm) were prepared, polymerized for 40 s from each side and the density D2 was measured. The polymerization shrinkage in % was calculated by ΔV = ((1 / D^2^) – (1 / D1)) x (1 / D1) x 100.

### Contact angle, SFE of solids and surface tension of active agents

Contact angles were determined with the sessile drop method (Phoenix-Alpha contact angle goniometer, Surface Electro Optics - SEO - Corporation, Suwon-City, Korea). Ten pictures of each drop were photographed (magnification 25-fold) in one second (CCD-camera: FireDragon, Toshiba-Teli Corporation, Tokyo, Japan) and analyzed (Image XP-software, Version 6.0 FW 012108, SEO Corporation).

To determine contact angles and SFE, ten discs (diameter 10 ± 0.1 mm, thickness 1 ± 0.1 mm) of each material were prepared and polymerized for 40 s from each side. Their surfaces were polished with fine and superfine polishing-discs (Super-Snap mini, Shofu Inc., Kyoto, Japan) for 1 minute each with a grinding pressure of 40 - 50 g and 10000 rpm (Endo-Mate TC, Nakanishi Inc., Tochigi, Japan). Contact angle Θ was measured one time on each specimen. Measurements with aqua dest. (laboratory product) were done before and after three weeks water storage at 37°C. Measurements with glycerol (LOT II1097071, Thermo Scientific, Rockford, IL, USA), ethylene glycol (LOT 3289749, Carl Roth GmbH & Co. KG, Karlsruhe, Germany) and diiodomethane (LOT S82251, Sigma Aldrich GmbH, Taufkirchen, Germany) were solely done after three weeks water storage at 37°C. SFE was calculated from the Θ measured after water storage. Apolar Lifshitz-van der Waals γ_L_^LW^, polar Lewis acid γ_L_^+^ and Lewis base γ_L_^-^ terms of the test liquids were taken from the literature [32] and the specimens’ total SFE γ_S_, their apolar term γ_S_^LW^, polar term γ_S_^AB^, acid term γ_S_^+^ and base term γ_S_^-^ were calculated according to the equation of van Oss et al. [35] by (cosΘ + 1) x γ_L_ = 2(√(γ_S_^LW^ γ_L_^LW^) + √(γ_S_^-^ γ_L_^+^) + √(γ_S_^+^ γ_L_^-^)) and with γ_S_^AB^ = 2√(γ_S_^+^ γ_S_^-^) (Image XP, Version 6.0 FW 072809, SEO Corporation). 

### Bacteria preparation

*Streptococcus sanguinis* (strain 20068), *Streptococcus oralis* (strain 20627, *Streptococcus mitis* (strain 12643), *Actinomyces viscosus* (strain 43329), and *Actinomyces naeslundii* (strain 17233, all strains from DSMZ GmbH, Braunschweig, Germany) were used to determine cell viability. Bacteria were exposed on agarplates and incubated for 48 hours at 37 °C (actinomyces under anaerobe conditions). Overnight cultures of the streptococci were established in sterile trypticase soy broth (Sigma-Aldrich Inc., St. Louis, MO, USA) supplemented with yeast extract (Carl Roth GmbH, Karlsruhe, Germany) at 37°C. Single colonies of the actinomyces were cultivated in sterile actinomyces broth (Vegiton, Sigma Aldrich GmbH) for 24 hours at 37 °C. The bacteria were harvested by centrifugation (1000 rpm, 10 min, 18 °C), washed twice with sterile 0.9 % NaCl solution and re-suspended again in sterile 0.9 % NaCl. Optical density of the suspensions was adjusted to 1iter at 600 nm (Smart Spec plus, Biorad Laboratories Inc., Hercules, CA, USA), which corresponded to a microbial concentration of 5 x 108 cells ml^-1^.

### Saliva preparation

Unstimulated human saliva was collected from ten healthy non-smoking subjects (age 28 - 58, mean 37.7 years). Approval from the ethics committee was obtained (Ethics committee of the Medical Faculty of Heinrich-Heine-University, Düsseldorf, Germany, internal study number: 2912). The participants gave their consent verbally, as they all were part of the academic faculty staff of the department. Documentation of the oral consent was done by making a list of prospective saliva donors. All donors who subsequently gave their oral consent were checked-off on this list. The oral consent was explicitly judged as adequate, and the need for written informed consent from the participants was waived by the ethics committee. The saliva samples were all mixed with each other, so that they could not be identified anymore after sampling. The saliva was centrifuged (30 min, 4500 rpm, 4 °C U, Universal 16R, Hettich GmbH, Tuttlingen, Germany) and the supernatant was sterile filtered (Millex-GV, 0.45 µm, PVDF, 3 mm, Millipore Inc., Billerica, MA, USA) and subsequently heated to 56 °C for 30 minutes (NeoBlock Heizer Mono I, neoLab Migge Laborbedarf-Vertriebs GmbH, Heidelberg, Germany). Finally the samples were pooled and stored at -20 °C. Prior to incubation samples were defrosted and 1:1 diluted with PBS (Dulbecco's Phosphate Buffered Saline modified, Sigma-Aldrich Inc.).

### Specimen preparation and incubation

Thirty disks (thickness: 1 ± 0.1 mm, diameter: 10 ± 0.1 mm) of each material were made and cured 40 s each side (Spectrum 800, Dentsply deTrey GmbH, Constance, Germany). The output of the curing device was routinely checked (bluephase Meter, Ivoclar Vivadent AG, Schaan, Liechtenstein). Irradiances of 931 ± 90 mW cm-^2^ were measured and no significant decrease of the output was observed. The cured specimens were stored for 14 days in water at 37°C, disinfected (Bacillol AF®, Bode Chemie, Hamburg, Germany) and then one side was wet-polished (sterile water) with fine and superfine polishing-discs (Super-Snap mini, Shofu Inc., Kyoto, Japan) for 1 min, with a grinding pressure of 40 - 50 g and 10000 rpm (Endo-Mate TC, Nakanishi Inc., Tochigi, Japan). Afterwards they were placed with the polished side up in a well (TC Test Plate 24 wells, Orange Scientific INC., Braine-l’Alleud, Belgium), incubated with 250 µl of the diluted saliva for 2 hours at 37 °C and finally washed twice with a sterile 0.9 % NaCl solution.

### Determination of cell viability (live/dead staining)

Three specimens (polished sides) for each material / bacterium / measurement point were used and incubated with 350 µl bacterial suspension for 8 hours or 24 hours, respectively, at 37 °C (streptococci at 5 % CO_2_, actinomyces at anaerobic conditions), then washed with 0.9 % NaCl solution and air-dried. Vital and non-vital cells were determined (LIVE/DEAD BacLight bacterial viability kit, Molecular Probes Inc., Eugene, OR, USA) by measuring the fluorescence emission (fluorescence microscope DM2500, Leica Microsystems Ltd., Wetzlar, Germany) on the blinded specimens. Fluorescent microscopic images of four randomly selected sites of each specimen (magnification 400 fold) were captured (digital camera DFC420C, Leica Microsystems Ltd.) with different fluorescence filter sets (N2.1 and I3, Leica Microsystems Ltd.). Vital and non-vital cells were calculated by counting pixel per colour (pixel count software, written by a stuff member of the department).

### Statistical analysis

Means and standard deviations were calculated. Normal distribution was tested by Kolmogoroff-Smirnoff-Test. Univariate ANOVA and post hoc Scheffé’s test were performed separately for each tested property and for each test material and bacterial subgroup (total, vital, non-vital). T-Test was used to identify differences of the contact angle between dry and wet storage and to find differences between the 8 hour and 24 hour bacteria counts. Multivariate ANOVA and post hoc Scheffé’s test were calculated to identify differences between the test materials’ SFEs (SPSS 15.0, SPSS, Chicago, IL, USA). Statistical significance for all tests was considered as p < 0.05.

## Results

Table 3 shows that material A had the lowest flexural modulus of all materials (all p > 0.0005). The water sorption decreased from ST to A, B and C (all p > 0.0005). The shrinkage of test materials did not differ from ST. The surface roughness of C was higher than of B (p = 0.023). Table 4 reports higher contact angles of the wet-stored materials A and C than of ST (all p < 0.0005). The total surface free energy γ_S_ of material B was lower than of ST and of C (all p < 0.0005) and the polar terms of the surface free energy γ_S_^AB^ of A and B were lower than of ST and of C (all p < 0.0005). The acid terms of the surface free energy γ_S_^+^ of materials A to C were higher than of ST (all p < 0.0005) and γ_S_^+^ of B was higher than of C (p = 0.026).

**Table 3:** Means and (standard deviations) of flexural strength, flexural modulus, water sorption, solubility and surface roughness R_a_, values are rounded to valid digits.

Material	Flexural strength [MPa]	Flexural modulus [MPa]	Water sorption [µg mm^-3^]	Solubility [µg mm^-3^]	R_a_[µm]	Shrinkage [vol.-%]
ST	101.6 (9.5)_1_	6525 (317)_1_	24.2 (1.6)	0.2 (0.8)_1_	0.15 (0.02)_1,2_	4.2 (0.9)_1_
A	85.7 (9.9)_1_	4516 (233)	21.3 (0.6)	0.1 (0.9)_1_	0.18 (0.06)_1,2_	2.9 (0.9)_1_
B	89.5 (9.6)_1_	6161 (242)_1_	18.8 (0.4)	-0.6 (0.2)_1_	0.13 (0.02)_1_	3.3 (0.2)_1_
C	100.9 (7.8)_1_	6618 (156)_1_	14.4 (0.3)	0.4 (0.3)_1_	0.24 (0.09)_2_	3.5 (0.4)_1_

The same subscript number within the columns indicates ***non*** significant differences (p < 0.05).

**Table 4:** Means and (standard deviations) of contact angles and surface free energies of dry stored and wet stored polished specimens, values are rounded to valid digits.

Material	Contact angle Θ [°]		Surface free energy, wet & polished [mJ m^-2^]				
	dry	wet	γ_S_	γ_S_^LW^	γ_S_^AB^	γ_S_^+^	γ_S_^-^
ST	66.2 (5.9)_1_	57.6 (4.3)_1_	43.2 (2.3)_1_	39.5 (2.4)_1_	3.7 (2.0)_1_	0.4 (0.2)	16.2 (2.8)_1_
A	78.3 (6.0)_1_	69.6 (7.6)_2_	39.3 (1.2)_1,2_	41.0 (2.5)_1_	-1.7 (2.3)_2_	1.5 (0.1)_1,2_	17.2 (4.5)_1_
B	72.6 (4.2)_1_	64.0 (5.5)_1,2_	38.7 (1.8)_2_	42.1 (2.2)_1_	-3.4 (2.5)_2_	1.8 (0.4)_2_	18.6 (6.3)_1_
C	75.0 (3.8)_1_	73.8 (3.6)_2_	42.8 (1.3)_1_	38.5 (2.6)_1_	4.3 (1.7)_1_	1.4 (0.3)_1_	11.3 (5.7)_1_

Underlined numbers indicate ***non*** significant differences between dry and wet storage. The same subscript number within the columns indicates ***non*** significant differences (p < 0.05).

γ_S_ = total surface free energy of the specimen

γ_S_^AB^ = polar term of the specimens surface free energy

γ_S_^LW^ = apolar term of the specimens surface free energy

γ_S_^+^ = acid term of the specimens surface free energy

γ_S_^-^ = base term of the specimens surface free energy

### Bacterial viability after 8 hours observation time

For the overall bacteria the total count of material A was higher but of B and C lower than of ST (all p < 0.0005). Materials A to C had less vital cells and A had more non-vital cells than ST (all p < 0.0005). Considering the individual species the total bacteria count was lower for *A. naeslundii* on material B (p = 0.015) and for *A. viscosus* and *S. sanguinis* on materials B and C than on ST (p < 0.0005 to p = 0.002). Higher total bacteria counts were observed for *A. viscosus* and *S. mitis* on material A (all p < 0.0005). Less vital cells were found for *A. viscosus*, *S.* oralis and *S. sanguinis* on materials A to C (p < 0.0005 to p = 0.031) but for *A. naeslundii* only on A (p = 0.011) and B (p = 0.003) and for *S. mitis* only on B and C than on ST (all p = 0.010). More non-vital bacteria were counted for each individual species on material A (all p < 0.0005). Materials A, B and C did not differ among each other in the vital cells of *S. sanguinis after* 8 hours. *S. oralis* had less vital cells (p = 0.002) on material A than on C.

### Bacterial viability after 24 hours observation time

For the overall bacteria consideration the total bacteria count decreased only on material C compared with 8 hours (p = 0.012). Materials B and C showed less vital (p < 0.0005 to p = 0.048), ST and B showed more non-vital (P = 0.001 and p = 0.017) but A showed less non-vital cells (P = 0.005). Considering the individual species less total *actinomyces* were found on materials B and C compared with ST (P = 0.006 to p = 0.014) This was also observed for *S. mitis* and *S. sanguinis* (p < 0.0005 to p = 0.039), but not for *S. oralis*. Less vital *actinomyces* also existed on A, B and C (p < 0.0005 to p = 0.003) but for *S. mitis* and *S. sanguinis* less vital cells existed only on B and C (p = 0.002 to p = 0.011) and for *S. oralis* only on A (p = 0.044). Less vital *actinomyces* lived on material C (p < 0.0005 to p = 0.002) but more total (p = 0.033) and more non-vital (p = 0.005) *A. naeslundii* were on material B. Total and non-vital *S. mitis* cells were only influenced by material A (p = 0.006 and p = 0.009) resulting in a decrease. The vital cells of *S. oralis* decreased on ST, B and C (p = 0.016 to p = 0.046) but of *S. sanguinis* only on ST and C (P = 0.024 and p = 0.002).

**Figure 3:**
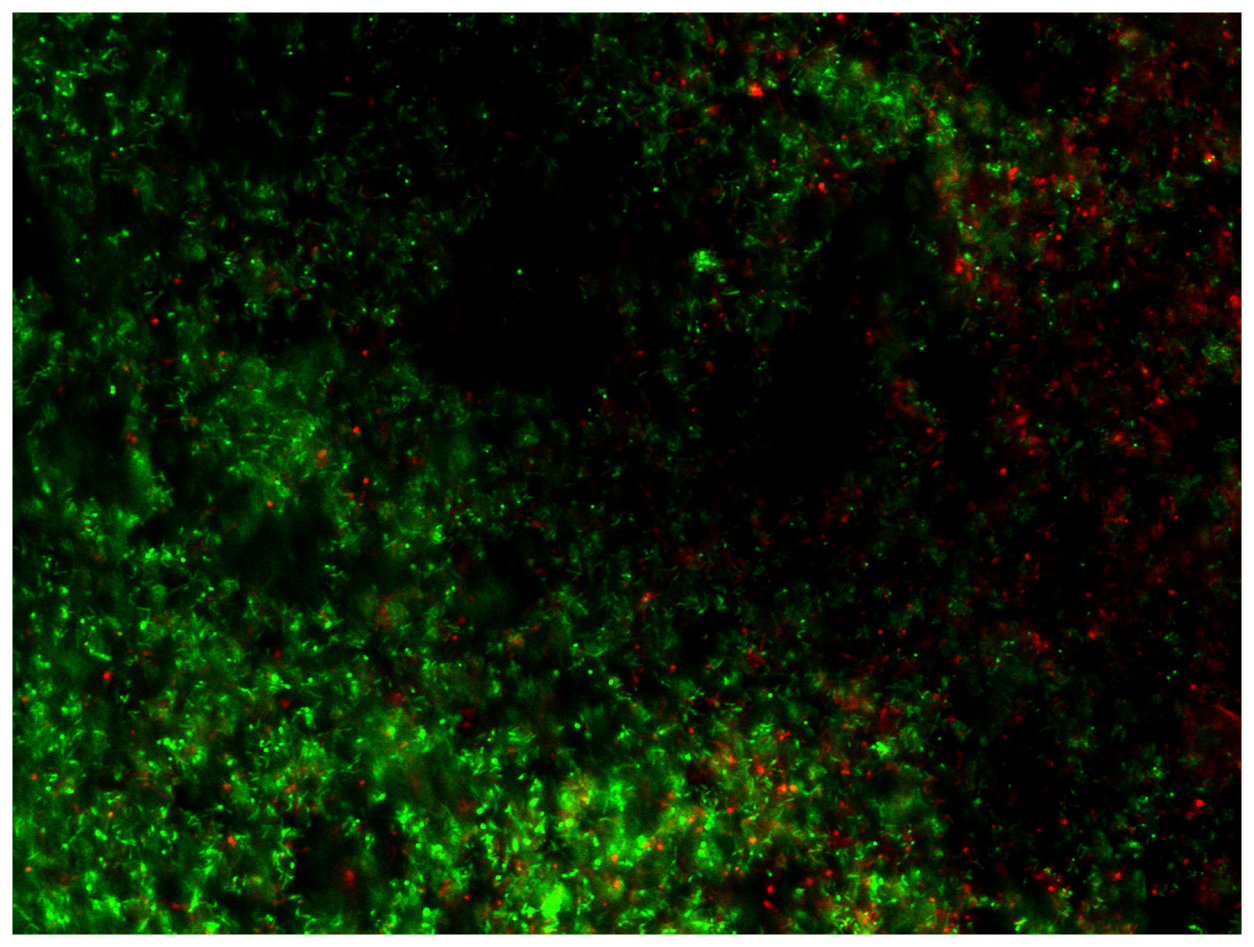
Superimposed fluorescence microscopic image (magnification 400 fold) of vital (green) and non-vital (red) colonization with *A. viscosus* on material ST after 24h.

**Figure 4:**
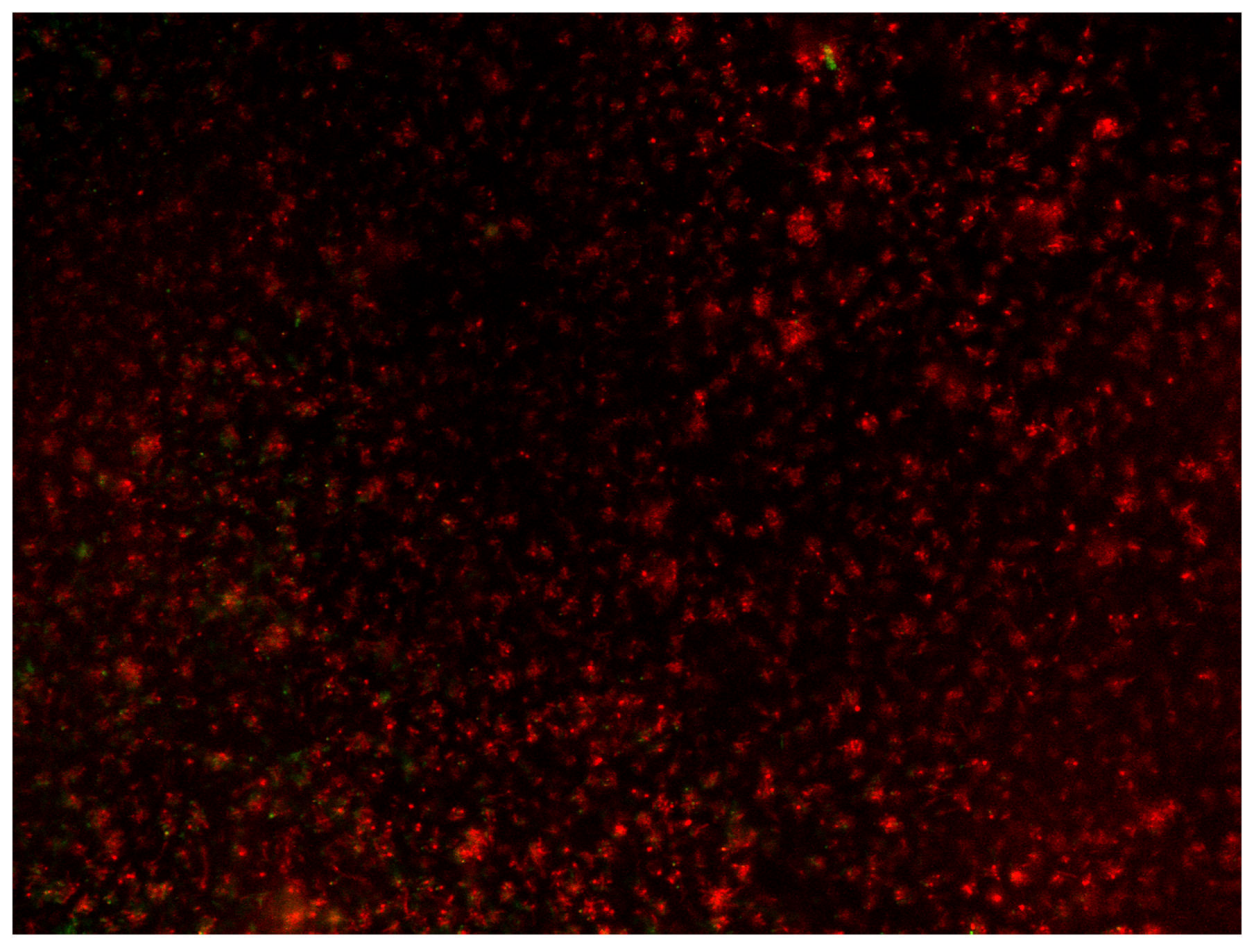
Superimposed fluorescence microscopic image (magnification 400 fold) of vital (green) and non-vital (red) colonization with *A. viscosus* on material A after 24h.

Materials A, .B and C did not differ among each other in the vital cells of the overall bacteria, of the actinomyces species, of S. mitis and S. oralis S. sanguinis showed more vital cells (p = 0.009) on materials A and C. Two examples of the vital fluorescence pictures can be seen in Figures 3 and 4.

## Discussion

The preparation of ST and the experimental dental composites [4,5], the determination of the materials properties [17,18,21,33,35-38] and of the cell viabilities [4,18,39-44] followed well-established methods and are exactly in accordance with the authors earlier publications [4,5]. It could be criticized, that the CFUs were not counted, but instead the number of pixels was assessed. Doing this only enables to compare the data between the same bacterial species, as the bacteria have different sizes. Nevertheless, we chose a computer-based assessment, as this method is more objective and less sensitive for mistakes. ST of the previous studies [4,5] was used and the experimental dental resins were formulated by substituting ST’s glass filler with the Poly-Irga delivery system, based on Poly-Pore, a novel highly effective sorption material [45], or by substituting the monomer matrix by Methacryl-Irga representing an antibacterial monomer [30-32]. An antibacterial effect of residual monomers was very unlikely because ST and the experimental materials did not differ in the type of the matrix or the filler but only in the addition of the Poly-Irga delivery system or the Methacryl-Irga. Although the degree of polymerization was not measured an antibacterial effect of residual monomers is very unlikely. ST and material A did not differ in the type of monomers but only in the amount of the loaded Poly-Pore replacing parts of the filler. The materials B and C contained the polymerizable Methacryl-Irga but due to the high irradiance of the light curing device and the very low solubility (0.2 ± 0.8) to 0.4 ± 0.3 µg mm-3) of all test materials and ST an optimal polymerization can be expected [4,5,37,38,46]. Furthermore, the test materials and ST did not differ in polymerization shrinkage what also indicated a good degree of conversion. These assumption are based on the literature which proved the relation between degree of conversion on solubility [47,48] or polymerization shrinkage [49-53]. The significance of surface roughness R_a_ on bacterial adherence was discussed thoroughly in the literature [18,36,54-59] and by the authors [4,5] and R_a_ ≤ 0.2 µm was judged to have negligible effect [18,56-58], therefore, based on the results of this investigation (Table 3), R_a_ is assumed not to be a factor. To mimic the salivary pellicle [60] the specimens were incubated in preparations of human saliva which was centrifuged and filtered to eliminate bacteria and heated to destroy lytic enzymes. Since the SFE of a saliva-coated bacterium was found to be same for original or heated (60 °C) saliva the heating process is considered to be allowed [26]. As already reported formerly [4] the typical protein spectrum appeared after gel electrophoresis which indicated the realistic simulation of in vivo pellicle formation. To investigate the experimental materials effect on cell viability at a very early stage of colonization, this investigation focused on the typical early colonizers of the oral biofilm [61]. As we expected our materials to have the highest impact at the beginning of bacterial colonization, we decided to investigate the early colonizers at first. It would be interesting for a following study to determine their effects on later colonizers like *S. mutans* or Lactobacilli, as these bacteria can also attach to the tooth/restoration surface directly. Based on the literature [62-64] and the found highly significantly results (see Results section) a reduced number of micrographs and an area of 0.99 mm^2^ were assumed to be appropriate for the present study. 

According to Table 3 the modifications of ST with the PolyPore delivery system or with Methacry-Irga did not influence the material properties unacceptably. All test materials achieved the minimum values for flexural strength, water sorption and solubility as requested by EN ISO 4049 [33]. Interestingly the water sorption even decreased from ST to material C. In this order the contact angle Θ increased what might explain this behaviour (Table 4). None of the materials showed total SFE γ_s_ < 30 mJ m^-2^ so that they were not really hydrophobic according to Vogler’s interpretation [65]. The polar terms γ_s_^AB^ of materials A and B were significantly lower than of ST which even suggests a higher hydrophilicity. 

The results of Table 5 prove the antibacterial effect of the modified composite resins A to C which had significantly less vital bacteria for the overall consideration as well as for most of the individual species and observation times. It is remarkable that the immobilized polymerizable Methacryl-Irga seems to be more effective (less vital cells) than the Irgasan which was set free from the delivery system (*S. mitis* for 8 and 24 hours and *S. sanguinis* for 24 hours). The results also show that amounts of 4 % (m/m) were adequate to reduce the number of vital cells significantly (material A and B) because no increased cell death was observed with 8 % (m/m) active agent in material C. *S. oralis* was the most resistant bacterium which did not differ in the vital cells from ST after 24 hours for material B and C and only material A had less vital cells for this observation time. This is the only case where the delivery system (material A) is more effective than Methacryl-Irga in 4 % (m/m) concentration (material B). Since materials A and B did not differ in their surface properties (Table 4) the higher sensibility of *S. oralis* against free Irgasan is the only plausible explanation.

**Table 5:** Means and (standard deviations) of total, vital bacterial and non-vital bacterial pixel-count after 8 h and 24 h observation time.

		Bacterial count [pixel x 10^6^]											
		Overall (n=60)		*A. naeslundii* (n=12)		*A. viscosus* (n=12)		*S. mitis* (n=12)		*S. oralis* (n=12)		*S. sanguis* (n=12)	
Material		8h	24h	8h	24h	8h	24h	8h	24h	8h	24h	8h	24h
ST	tot	5.1 (3.3)	6.0 (5.1)_1_	5.5 (2.6)_1,2_	6.9 (4.4)_1_	4.7 (2.7)	6.6 (5.6)_1_	2.1 (2.1)_1_	2.1 (2.2)_1_	6.9 (2.7)_1,2_	6.6 (4.8)_1_	6.5 (4.1)_1_	7.8 (6.2)_1_
	vit	3.8 (3.1)	3.7 (3.7)	2.8 (2.2)_1_	3.9 (3.0)	3.5 (2.6)	5.7 (4.9)	1.3 (1.5)_1_	1.6 (2.2)_1_	5.5 (2.8)	3.7 (3.3)_1_	6.0 (3.7)	3.5 (3.6)_1_
	non	1.4 (1.6)_1_	2.3 (2.4)_1_	2.7 (2.3)_1_	3.1 (1.9)_1_	1.2 (1.3)_1_	1.0 (0.9)_1_	0,8 (1.0)_1_	0.6 (0.4)_1_	1.5 (0.9)_1_	2.9 (2.5)_1_	0.6 (1.3)_1_	4.3 (3.3)_1_
A	tot	7.9 (3.3)	7.1 (4.0)_1_	8.2 (4.1)_1_	7.8 (3.6)_1_	10.0 (1.1)	8.1 (3.8)_1_	6.5 (3.7)	3.2 (2.0)_1_	7.7 (3.2)_2_	8.4 (4.1)_1_	6.9 (2.8)_1_	8.2 (3.9)_1_
	vit	1.0 (1.1)_1_	1.5 (1.6)_1_	0.8 (1.1)_2_	1.1 (1.1)_1_	0.5 (0.3)_1_	0.6 (0.6)_1_	0.9 (0.5)_1,2_	0.6 (0.4)_1,2_	0.6 (0.5)_1_	1.4 (1.2)_2_	2.1 (1.6)_1_	3.6 (1.8)_1_
	non	6.9 (3.4)	5.7 (3.4)	7.5 (3.7)	6.7 (2.8)	9.4 (1.2)	7.5 (3.5)	5.6 (3.7)	2.6 (2.0)	7.2 (3.0)	7.0 (3.7)	4.7 (3.1)	4.6 (2.5)_1_
B	tot	1.7 (1.8)_1_	1.9 (1.8)_2_	1.9 (1.0)_3_	3.1 (1.2)_2_	1.2 (0.9)_1_	1.3 (1.1)_2_	0.2 (0.07)_1_	0.2 (0.1)_2_	4.7 (1.1)_1_	4.3 (1.2)_1_	0.3 (0.3)_2_	0.5 (0.4)_2_
	vit	0.8 (0.9)_1_	0.6 (0.8)_1_	0.5 (0.4)_2_	0.4 (0.3)_1_	0.6 (0.3)_1_	0.7 (0.8)_1_	0.05 (0.03)_2_	0.2 (0.1)_2_	2.4 (0.7)_1,2_	1.6 (0.8)_1,2_	0.3 (0.3)_1_	0.1 (0.1)_2_
	non	0.9 (1.0)_1_	1.3 (1.3)_1_	1.4 (0.6)_1_	2.7 (1.1)_1_	0.6 (0.7)_1_	0.7 (0.6)_1_	0.2 (0.06)_1_	0.05 (0.04)_1_	2.3 (0.7)_1,2_	2.7 (0.8)_1_	0.08 (0.03)_1_	0.4 (0.4)_2_
C	tot	2.9 (3.0)_1_	2.2 (2.0)_2_	4.7 (2.2)_2,3_	2.6 (0.9)_2_	2.0 (0.5)_1_	1.8 (1.2)_2_	0.3 (0.1)_1_	0.3 (0.1)_2_	7.5 (1.3)_2_	5.2 (2.0)_1_	0.2 (0.1)_2_	1.1 (0.5)_2_
	vit	1.4 (1.5)_1_	0.7 (1.0)_1_	1.9 (1.4)_1,2_	0.4 (0.4)_1_	1.3 (0.4)_1_	0.3 (0.3)_1_	0.07 (0.04)_2_	0.05 (0.02)_2_	3.4 (1.1)_2_	2.1 (1.3)_1,2_	0.05 (0.04)_1_	0.4 (0.3)_2_
	non	1.6 (1.7)_1_	1.5 (1.3)_1_	2.7 (1.1)_1_	2.1 (0.7)_1_	0.6 (0.3)_1_	1.5 (1.1)_1_	0.2 (0.1)_1_	0.3 (0.1)_1_	4.0 (0.7)_2_	3.1 (1.2)_1_	0.2 (0.1)_1_	0.6 (0.4)_2_

Values are rounded to valid digits. Underlined numbers within the rows indicate ***non*** significant differences between the 8 h and 24 h observation period of each bacteria group. The same superscript number within the columns indicates ***non*** significant differences between total, vital or non-vital bacterial pixel-count, respectively (p > 0.05).

tot = total, vit = vital, non = non-vital

Considering the total bacteria counts it is very striking that significantly less cells were found on materials B and C than on ST and mostly also on material A. Again *S. oralis* was the only exception from this observation. This means that *S. oralis* was not only significantly more resistant against Irgasan but also has very strong adhesive forces which were not reduced by the materials’ chemistry. Material A which provides free Irgasan due to the PolyPore delivery system did not show less total bacteria for none of the tested species but sometimes even a higher total bacteria count was calculated. This behaviour connected with the powerful antibacterial effect (less vital cells than ST) strongly indicated that an antibacterial effect did not necessarily diminish bacterial adhesive forces. As shown by the results for materials B and C the chemistry of the antibacterial ingredient needs also to be considered. Although nearly insoluble in water, the surface concentration of the free Irgasan provided by the delivery system might be reduced during the manipulation processes of the specimens. This could not be happen with the immobilized polymerizable Methacryl-Irga.

An influence of SFE on bacterial adherence was not clearly found in this study. As already discussed previously [4] a high polar term γ_s_^AB^ was reported to create strong bacterial adhesion [21,66], which implies that that low γ_s_^AB^ might lessen bacterial adhesive forces. Considering the total bacteria counts which might be an indicator for bacterial adhesion no relation was detected between the number of total bacteria and γ_s_^AB^. Table 4 in combination with Table 5 shows that material A and B did not differ in γ_s_^AB^ but material B had significantly less total bacteria for all species with the exception of *S. oralis* and material C with the same γ_s_^AB^ than ST had less total cells than ST and did not differ from B with lower γ_s_^AB^. The differences of materials B and C for γ_s_^AB^ were not surprising because Irgasan is a very hydrophobic substance and its concentration was twice as much in material C than in material B. It is hardly to explain why γ_s_^AB^ of material A and B was highly significantly lower than of ST. It might be possible that the polar groups of Irgasan (Cl- and O-atoms) turned away from the hydrophobic parts of the matrix and levelled to the hydrophilic environment. 

The much higher Methacryl-Irga concentration of material C might have overcompensated this effect which explains the increased γ_s_^AB^.

The present study is certainly limitated by the fact that the antibacterial effects needs not only to be investigated in-vitro but also in-vivo. Furthermore, other antibacterial substance or combinations thereof should be included in the delivery system. To test combinations of the present antibacterial monomers or delivery systems with the bacteria-repellent formulations of previous investigations [4,5] would certainly be of great interest.

## Conclusion

Experimental dental resin composites modified with small quantities of a novel antibacterially doped delivery system or with an antibacterial monomer were described that provided acceptable physical properties and good antibacterial effectiveness. The sorption material being part of the delivery system can be used as a vehicle for any other, perhaps even a more effective, active agent. Based on the results of the study the null hypothesis has to be rejected in total.

## References

[1] Konishi N, Torii Y, Kurosaki A, Takatsuka T, Itota T et al. (2003) Confocal laser scanning microscopic analysis of early plaque formed on resin composite and human enamel. J Oral Rehabil 30: 790-795. doi:10.1046/j.1365-2842.2003.01129.x. PubMed: 12880401. 12880401

[2] Persson A, Claesson R, Van Dijken JW (2005) Levels of mutans streptococci and lactobacilli in plaque on aged restorations of an ion-releasing and a universal hybrid composite resin. Acta Odontol Scand 63: 21-25. PubMed: 16095058. 16095058 10.1080/00016350510019649

[3] Persson A, Lingstrom P, van Dijken JW (2005) Effect of a hydroxyl ion-releasing composite resin on plaque acidogenicity. Caries Res 39: 201-206. doi:10.1159/000084799. PubMed: 15914982. 15914982

[4] Rüttermann S, Trellenkamp T, Bergmann N, Raab WH, Ritter H et al. (2011) A new approach to influence contact angle and surface free energy of resin-based dental restorative materials. Acta Biomater 7: 1160-1165. doi:10.1016/j.actbio.2010.10.002. PubMed: 20933616. 20933616

[5] Rüttermann S, Bergmann N, Beikler T, Raab WH, Janda R (2012) Bacterial viability on surface-modified resin-based dental restorative materials. Arch Oral Biol 57: 1512-1521. doi:10.1016/j.archoralbio.2012.05.005. PubMed: 22673754. 22673754

[6] Thomas RZ, van der Mei HC, van der Veen MH, de Soet JJ, Huysmans MC (2008) Bacterial composition and red fluorescence of plaque in relation to primary and secondary caries next to composite: an in situ study. Oral Microbiol Immunol 23: 7-13. PubMed: 18173792. 18173792 10.1111/j.1399-302X.2007.00381.x

[7] van Dijken JW, Kalfas S, Litra V, Oliveby A (1997) Fluoride and mutans streptococci levels in plaque on aged restorations of resin-modified glass ionomer cement, compomer and resin composite. Caries Res 31: 379-383. doi:10.1159/000262422. PubMed: 9286522. 9286522

[8] Wiegand A, Buchalla W, Attin T (2007) Review on fluoride-releasing restorative materials--fluoride release and uptake characteristics, antibacterial activity and influence on caries formation. Dent Mater 23: 343-362. doi:10.1016/j.dental.2006.01.022. PubMed: 16616773. 16616773

[9] Ahn SJ, Lee SJ, Kook JK, Lim BS (2009) Experimental antimicrobial orthodontic adhesives using nanofillers and silver nanoparticles. Dent Mater 25: 206-213. doi:10.1016/j.dental.2008.06.002. PubMed: 18632145. 18632145

[10] Gyo M, Nikaido T, Okada K, Yamauchi J, Tagami J et al. (2008) Surface response of fluorine polymer-incorporated resin composites to cariogenic biofilm adherence. Appl Environ Microbiol 74: 1428-1435. doi:10.1128/AEM.02039-07. PubMed: 18192415. 18192415 PMC2258632

[11] Namba N, Yoshida Y, Nagaoka N, Takashima S, Matsuura-Yoshimoto K et al. (2009) Antibacterial effect of bactericide immobilized in resin matrix. Dent Mater 25: 424-430. doi:10.1016/j.dental.2008.08.012. PubMed: 19019421. 19019421

[12] Li F, Chen J, Chai Z, Zhang L, Xiao Y et al. (2009) Effects of a dental adhesive incorporating antibacterial monomer on the growth, adherence and membrane integrity of Streptococcus mutans. J Dent 37: 289-296. doi:10.1016/j.jdent.2008.12.004. PubMed: 19185408. 19185408

[13] Imazato S, Ohmori K, Russell RR, McCabe JF, Momoi Y et al. (2008) Determination of bactericidal activity of antibacterial monomer MDPB by a viability staining method. Dent Mater J 27: 145-148. doi:10.4012/dmj.27.145. PubMed: 18309624. 18309624

[14] Imazato S, Ebi N, Takahashi Y, Kaneko T, Ebisu S et al. (2003) Antibacterial activity of bactericide-immobilized filler for resin-based restoratives. Biomaterials 24: 3605-3609. doi:10.1016/S0142-9612(03)00217-5. PubMed: 12809790. 12809790

[15] Beyth N, Yudovin-Farber I, Bahir R, Domb AJ, Weiss EI (2006) Antibacterial activity of dental composites containing quaternary ammonium polyethylenimine nanoparticles against Streptococcus mutans. Biomaterials 27: 3995-4002. doi:10.1016/j.biomaterials.2006.03.003. PubMed: 16564083. 16564083

[16] Beyth N, Houri-Haddad Y, Baraness-Hadar L, Yudovin-Farber I, Domb AJ et al. (2008) Surface antimicrobial activity and biocompatibility of incorporated polyethylenimine nanoparticles. Biomaterials 29: 4157-4163. doi:10.1016/j.biomaterials.2008.07.003. PubMed: 18678404. 18678404

[17] Quirynen M, Marechal M, Busscher HJ, Weerkamp AH, Arends J et al. (1989) The influence of surface free-energy on planimetric plaque growth in man. J Dent Res 68: 796-799. doi:10.1177/00220345890680050801. PubMed: 2715472. 2715472

[18] Quirynen M, Marechal M, Busscher HJ, Weerkamp AH, Darius PL et al. (1990) The influence of surface free energy and surface roughness on early plaque formation. An in vivo study in man. J Clin Periodontol 17: 138-144. doi:10.1111/j.1600-051X.1990.tb01077.x. PubMed: 2319000. 2319000

[19] Buergers R, Schneider-Brachert W, Hahnel S, Rosentritt M, Handel G (2009) Streptococcal adhesion to novel low-shrink silorane-based restorative. Dent Mater 25: 269-275. doi:10.1016/j.dental.2008.07.011. PubMed: 18768217. 18768217

[20] Rupp F, Axmann D, Ziegler C, Geis-Gerstorfer J (2002) Adsorption/desorption phenomena on pure and Teflon AF-coated titania surfaces studied by dynamic contact angle analysis. J Biomed Mater Res 62: 567-578. doi:10.1002/jbm.10198. PubMed: 12221705. 12221705

[21] Knorr SD, Combe EC, Wolff LF, Hodges JS (2005) The surface free energy of dental gold-based materials. Dent Mater 21: 272-277. doi:10.1016/j.dental.2004.06.002. PubMed: 15705434. 15705434

[22] Hannig M, Kriener L, Hoth-Hannig W, Becker-Willinger C, Schmidt H (2007) Influence of nanocomposite surface coating on biofilm formation in situ. J Nanosci Nanotechnol 7: 4642-4648. PubMed: 18283856. 18283856

[23] Hahnel S, Rosentritt M, Handel G, Bürgers R (2009) Surface characterization of dental ceramics and initial streptococcal adhesion in vitro. Dent Mater 25: 969-975. doi:10.1016/j.dental.2009.02.003. PubMed: 19278720. 19278720

[24] Hahnel S, Rosentritt M, Bürgers R, Handel G (2008) Surface properties and in vitro Streptococcus mutans adhesion to dental resin polymers. J Mater Sci Mater Med 19: 2619-2627. doi:10.1007/s10856-007-3352-7. PubMed: 18197372. 18197372

[25] Hahnel S, Rosentritt M, Handel G, Bürgers R (2008) Influence of saliva substitute films on initial Streptococcus mutans adhesion to enamel and dental substrata. J Dent 36: 977-983. doi:10.1016/j.jdent.2008.08.004. PubMed: 18789569. 18789569

[26] Weerkamp AH, van der Mei HC, Busscher HJ (1985) The surface free energy of oral streptococci after being coated with saliva and its relation to adhesion in the mouth. J Dent Res 64: 1204-1210. doi:10.1177/00220345850640100501. PubMed: 3861650. 3861650

[27] Kapoor M, Reddy CC, Krishnasastry MV, Surolia N, Surolia A (2004) Slow-tight-binding inhibition of enoyl-acyl carrier protein reductase from Plasmodium falciparum by triclosan. Biochem J 381: 719-724. doi:10.1042/BJ20031821. PubMed: 15086316. 15086316 PMC1133881

[28] Lupu R, Menendez JA (2006) Pharmacological inhibitors of Fatty Acid Synthase (FASN)--catalyzed endogenous fatty acid biogenesis: a new family of anti-cancer agents? Curr Pharm Biotechnol 7: 483-493. doi:10.2174/138920106779116928. PubMed: 17168665. 17168665

[29] Sharma S, Ramya TN, Surolia A, Surolia N (2003) Triclosan as a systemic antibacterial agent in a mouse model of acute bacterial challenge. Antimicrob Agents Chemother 47: 3859-3866. doi:10.1128/AAC.47.12.3859-3866.2003. PubMed: 14638495. 14638495 PMC296231

[30] Choi S-b, Jepperson J, Jarabek L, Thomas J, Chisholm B et al. (2007) Novel Approach to Anti-Fouling and Fouling-Release Marine Coatings Based on Dual-Functional Siloxanes. Macromol Symp 249-250: 660-667. doi:10.1002/masy.200750452.

[31] Oh ST, Ha CS, Cho WJ (1994) Synthesis and biocidal activities of polymer. III. Bactericical activity of homopolymer of AcDP and copolymer of acdp with St. Journal of Applied. J Polym Sci 54: 859-866.

[32] Oh ST, Han SH, Ha CS, Cho WJ (1996) Synthesis and biocidal activities of polymer. IV. Antibacterial activity and hydrolysis of polymers containing diphenyl ether. J Appl Polym Sci 59: 1871-1878. doi:10.1002/(SICI)1097-4628(19960321)59:12.

[33] En ISO 4049: Dentistry - Polymer-based filling, restorative and luting materials

[34] DIN EN ISO 4288: Rules and Procedures for Assessment of Surface Texture.

[35] van Oss CJ, Chaudhury MK, Good RJ (1988) Interfacial Lifshitz-van der Waals and polar interactions in macroscopic systems. Chem Rev 88: 927-941. doi:10.1021/cr00088a006.

[36] Quirynen M, Bollen CML (1995) The influence of surface roughness and surface-free energy on supra- and subgingival plaque formation in man. A review of the literature. J Clin Periodontol 22: 1-14. PubMed: 7706534. 7706534 10.1111/j.1600-051x.1995.tb01765.x

[37] Janda R, Roulet JF, Latta M, Rüttermann S (2006) The effects of thermocycling on the flexural strength and flexural modulus of modern resin-based filling materials. Dent Mater 22: 1103-1108. doi:10.1016/j.dental.2005.09.005. PubMed: 16406120. 16406120

[38] Janda R, Roulet JF, Latta M, Rüttermann S (2007) Water sorption and solubility of contemporary resin-based filling materials. J Biomed Mater Res B Appl Biomater 82: 545-551. PubMed: 17285606. 17285606 10.1002/jbm.b.30760

[39] Al-Ahmad A, Follo M, Selzer AC, Hellwig E, Hannig M et al. (2009) Bacterial colonization of enamel in situ investigated using fluorescence in situ hybridization. J Med Microbiol 58: 1359-1366. doi:10.1099/jmm.0.011213-0. PubMed: 19528150. 19528150

[40] Al-Ahmad A, Wunder A, Auschill TM, Follo M, Braun G et al. (2007) The in vivo dynamics of Streptococcus spp., Actinomyces naeslundii, Fusobacterium nucleatum and Veillonella spp. in dental plaque biofilm as analysed by five-colour multiplex fluorescence in situ hybridization. J Med Microbiol 56: 681-687. doi:10.1099/jmm.0.47094-0. PubMed: 17446294. 17446294

[41] Hannig C, Hannig M, Rehmer O, Braun G, Hellwig E et al. (2007) Fluorescence microscopic visualization and quantification of initial bacterial colonization on enamel in situ. Arch Oral Biol 52: 1048-1056. doi:10.1016/j.archoralbio.2007.05.006. PubMed: 17603998. 17603998

[42] ten Cate JM (2006) Biofilms, a new approach to the microbiology of dental plaque. Odontology 94: 1-9. doi:10.1007/s10266-006-0063-3. PubMed: 16998612. 16998612

[43] Jung DJ, Al-Ahmad A, Follo M, Spitzmüller B, Hoth-Hannig W et al. (2010) Visualization of initial bacterial colonization on dentine and enamel in situ. J Microbiol Methods 81: 166-174. doi:10.1016/j.mimet.2010.03.002. PubMed: 20211207. 20211207

[44] Hannig C, Follo M, Hellwig E, Al-Ahmad A (2010) Visualization of adherent micro-organisms using different techniques. J Med Microbiol 59: 1-7. doi:10.1099/jmm.0.015420-0. PubMed: 19815663. 19815663

[45] Sojka MS (1998) Precipitation Polymerization process for producing an oil adsorbent polymer capable of entrapping solid particles and liquids and the product thereof. US Patent No US5830960

[46] Rüttermann S, Krüger S, Raab WH, Janda R (2007) Polymerization shrinkage and hygroscopic expansion of contemporary posterior resin-based filling materials-A comparative study. J Dent 35: 806-813. doi:10.1016/j.jdent.2007.07.014. PubMed: 17826883. 17826883

[47] da Silva EM, Almeida GS, Poskus LT, Guimarães JG (2008) Relationship between the degree of conversion, solubility and salivary sorption of a hybrid and a nanofilled resin composite. J Appl Oral Sci 16: 161-166. PubMed: 19089210. 19089210 10.1590/S1678-77572008000200015 PMC4327638

[48] Gonçalves L, Filho JD, Guimarães JG, Poskus LT, Silva EM (2008) Solubility, salivary sorption and degree of conversion of dimethacrylate-based polymeric matrixes. J Biomed Mater Res B Appl Biomater 85: 320-325. PubMed: 17973246. 17973246 10.1002/jbm.b.30949

[49] Sideridou ID, Karabela MM, Micheliou CN, Karagiannidis PG, Logothetidis S (2009) Physical properties of a hybrid and a nanohybrid dental light-cured resin composite. J Biomater Sci Polym Ed 20: 1831-1844 doi:10.1163/156856208X386435. PubMed: 19793442. 19793442

[50] Cho E, Sadr A, Inai N, Tagami J (2011) Evaluation of resin composite polymerization by three dimensional micro-CT imaging and nanoindentation. Dent Mater 27: 1070-1078. doi:10.1016/j.dental.2011.07.008. PubMed: 21820729. 21820729

[51] Sharifi S, Mirzadeh H, Imani M, Atai M, Ziaee F (2008) Photopolymerization and shrinkage kinetics of in situ crosslinkable N-vinyl-pyrrolidone/poly(epsilon-caprolactone fumarate) networks. J Biomed Mater Res A 84: 545-556. PubMed: 17647285. 17647285 10.1002/jbm.a.31384

[52] Feng L, Suh BI (2006) The effect of curing modes on polymerization contraction stress of a dual cured composite. J Biomed Mater Res B Appl Biomater 76: 196-202. PubMed: 16047326. 16047326 10.1002/jbm.b.30355

[53] Silikas N, Eliades G, Watts DC (2000) Light intensity effects on resin-composite degree of conversion and shrinkage strain. Dent Mater 16: 292-296. doi:10.1016/S0109-5641(00)00020-8. PubMed: 10831785. 10831785

[54] Aykent F, Yondem I, Ozyesil AG, Gunal SK, Avunduk MC et al. (2010) Effect of different finishing techniques for restorative materials on surface roughness and bacterial adhesion. J Prosthet Dent 103: 221-227. doi:10.1016/S0022-3913(10)60034-0. PubMed: 20362765. 20362765

[55] Beyth N, Bahir R, Matalon S, Domb AJ, Weiss EI (2008) Streptococcus mutans biofilm changes surface-topography of resin composites. Dent Mater 24: 732-736. doi:10.1016/j.dental.2007.08.003. PubMed: 17897707. 17897707

[56] Bollen CM, Lambrechts P, Quirynen M (1997) Comparison of surface roughness of oral hard materials to the threshold surface roughness for bacterial plaque retention: a review of the literature. Dent Mater 13: 258-269. doi:10.1016/S0109-5641(97)80038-3. PubMed: 11696906. 11696906

[57] Bollen CM, Papaioanno W, Van Eldere J, Schepers E, Quirynen M et al. (1996) The influence of abutment surface roughness on plaque accumulation and peri-implant mucositis. Clin Oral Implants Res 7: 201-211. doi:10.1034/j.1600-0501.1996.070302.x. PubMed: 9151584. 9151584

[58] Quirynen M, Bollen CM, Papaioannou W, Van Eldere J, van Steenberghe D (1996) The influence of titanium abutment surface roughness on plaque accumulation and gingivitis: short-term observations. Int J Oral Maxillofac Implants 11: 169-178. PubMed: 8666447. 8666447

[59] Teughels W, Van Assche N, Sliepen I, Quirynen M (2006) Effect of material characteristics and/or surface topography on biofilm development. Clin Oral Implants Res 17 Suppl 2: 68-81. doi:10.1111/j.1600-0501.2006.01353.x. PubMed: 16968383. 16968383

[60] Rosan B, Lamont RJ (2000) Dental plaque formation. Microbes Infect 2: 1599-1607. doi:10.1016/S1286-4579(00)01316-2. PubMed: 11113379. 11113379

[61] Kolenbrander PE, Palmer RJ Jr., Periasamy S, Jakubovics NS (2010) Oral multispecies biofilm development and the key role of cell-cell distance. Nat Rev Microbiol 8: 471-480. doi:10.1038/nrmicro2381. PubMed: 20514044. 20514044

[62] Buergers R, Rosentritt M, Handel G (2007) Bacterial adhesion of Streptococcus mutans to provisional fixed prosthodontic material. J Prosthet Dent 98: 461-469. doi:10.1016/S0022-3913(07)60146-2. PubMed: 18061740. 18061740

[63] Buergers R, Schneider-Brachert W, Hahnel S, Rosentritt M, Handel G (2009) Streptococcal adhesion to novel low-shrink silorane-based restorative. Dent Mater 25: 269-275. doi:10.1016/j.dental.2008.07.011. PubMed: 18768217. 18768217

[64] Gosau M, Hahnel S, Schwarz F, Gerlach T, Reichert TE et al. (2010) Effect of six different peri-implantitis disinfection methods on in vivo human oral biofilm. Clin Oral Implants Res 21: 866-872. PubMed: 20666798. 20666798 10.1111/j.1600-0501.2009.01908.x

[65] Vogel BS, Wildung MR, Vogel G, Croteau R (1996) Abietadiene synthase from grand fir (Abies grandis). cDNA isolation, characterization, and bacterial expression of a bifunctional diterpene cyclase involved in resin acid biosynthesis. J Biol Chem 271: 23262-23268. doi:10.1074/jbc.271.38.23262. PubMed: 8798524. 8798524

[66] Lee SP, Lee SJ, Lim BS, Ahn SJ (2009) Surface characteristics of orthodontic materials and their effects on adhesion of mutans streptococci. Angle Orthod 79: 353-360. doi:10.2319/021308-88.1. PubMed: 19216592. 19216592

