# Correction: Bacterial Viability and Physical Properties of Antibacterially Modified Experimental Dental Resin Composites

**DOI:** 10.1371/journal.pone.0126198

**Published:** 2015-05-01

**Authors:** The PLOS ONE Staff

There is a section missing from the Materials and Methods. The section should appear between the “Manufacturing of the delivery system” and the “Analytical data” subsections. The publisher apologizes for this error. The missing section is:

## Synthesis of Methacryl-Irga [30–32]

23.5 g (81.2 m mol) Irgasan and 7.78 g (77 m mol) triethylamin (99%, Acros Organics, Geel, Belgium) were dissolved in 350 ml tetrahydrofuran (stabilized, Acros Organics), extra dried over molecular sieve, water content < 50 ppm in a 1-l three-necked flask with reflux condenser. The solution was cooled to 0°C and 7.71 g (73.8 m mol) methacryloyl chloride (> 97%, Sigma Aldrich GmbH, Taufkirchen, Germany) was dropped thereto within 90 min while stirring. After stirring overnight at room temperature, the reaction mixture was filtered to remove precipitated triethylamin hydrochloride. Phenothiazine (Sigma Aldrich) was added and tetrahydrofuran was distilled off. The crude product was dissolved in 100 ml n-hexane (Sigma Aldrich), washed with 5% sodium bicarbonate solution (3 x 40 ml), then with aqua dest. (4 x 50 ml). The organic phase was filtered over basic aluminium oxide and distilled. Yield: 22.36 g (63 m mol, 81.8%).

^1^H-NMR and ^13^C-NMR (Bruker Advance DRX 500 spectrometer, Bruker BioSpin GmbH, Rheinstetten, Germany) were performed; 500.13 MHz for proton and 125.77 MHz for carbon in CDCl_3_. The *δ*-scale relative to tetramethylsilan was calibrated to the solvent value δ = 7.24 ppm for CDCl_3_. IR spectra were recorded (Nicolet 5 SXB FT-IR Spectrometer, Thermo Scientific, Karlsruhe, Germany) with an attenuated total reflection (ATR) unit. Structure analysis was done with gas chromatography-mass spectrometry (GC/MS, Thermo Finnigan Trace DSQ, Dual-Stage Quadrupole, Thermo Scientific) and electron ionization (EI).
